# In Silico Characterization of Desert Locust–Derived Antimicrobial Peptides Against Drug‐Resistant Bacteria

**DOI:** 10.1155/sci5/7657843

**Published:** 2026-10-28

**Authors:** Peter M. Mwangi, Lilly C. Bebora, James K. Gathumbi, Xavier Cheseto, Cynthia M. Mudalungu, Segenet Kelemu, Chrysantus M. Tanga

**Affiliations:** ^1^ International Centre of Insect Physiology and Ecology (icipe) P.O. Box 30772-00100 Nairobi Kenya icipe.org; ^2^ Faculty of Veterinary Medicine Department of Veterinary Pathology, Microbiology, and Parasitology University of Nairobi P.O. Box 29053–00625 Nairobi Kenya uonbi.ac.ke; ^3^ Department of Chemistry and Materials Science The Technical University of Kenya (TUK) P.O. Box 52428-00100 Nairobi Kenya

**Keywords:** antibiotics, antimicrobial activity, desert locust, hemolysis, microbes, natural peptides

## Abstract

The critical need to find solutions to antibiotic resistance in pathogenic bacteria is becoming increasingly pronounced globally. In response to the urgent problems posed by the current public health challenges, our study adds to the growing body of knowledge by extracting insect peptides using acidified solvents, identifying the peptides from immune‐challenged and nonimmune‐challenged *Schistocerca gregaria* nymphs, and evaluating their antimicrobial activities against 9 clinical pathogens. A total of 96 unique peptides were identified, 91% from immune‐challenged nymphs. Three specific antimicrobial peptides (AMPs) were common in immune‐challenged and nonimmune‐challenged individuals. Eleven and 27 peptides were categorized as high‐ and moderate‐confidence AMPs, respectively, with sequence homologies ranging between 33% and 67%. The minimum inhibitory concentration (MIC) of peptide extract from immune‐challenged nymphs ranged between 5 and 25 μg/mL, compared to 7.9–100 μg/mL from nonimmune‐challenged nymphs. The lowest MIC values (< 8 μg/mL) were observed against *Staphylococcus aureus*, *Enterococcus faecalis, Listeria monocytogenes*, *Escherichia coli,* and *Streptococcus agalactiae*. It was observed that 87.5% of the AMP extracts had lower MIC values against *Strep. agalactiae* and *Pseudomonas aeruginosa* compared to tetracycline. The hemolytic activities due to peptide extracts from nonimmune‐challenged (SPE‐A) and immune‐challenged (SPE‐B) locust nymphs were < 5%. Our work underscores insects’ promise as sustainable AMP contributors, offering recommendations for further research to unlock their full potential in the design of new antimicrobial agents to overcome bacterial drug resistance.

## 1. Introduction

The surging tide of antimicrobial resistance (AMR) stands as a great challenge to human and animal health [1, 2]. The widespread and indiscriminate use of antibiotics in humans, animals, and plants has led to an increase in pathogenic bacteria that are resistant to them. This has, in turn, negatively affected the efficacy of the conventional antimicrobials against resistant bacteria. The management of illnesses brought on by these resistant strains has also become difficult and costly [2, 3]. In 2019, bacterial AMR was directly responsible for almost 1.3 million global fatalities and close to 1.14 million global deaths in 2021 [2, 4, 5]. Therefore, there is an urgent need to come up with other antimicrobial alternatives that will tackle this global menace.

Antimicrobial peptides (AMPs) can act as alternatives to conventional antibiotics. Some have been approved by the FDA, while clinical trials of others are still ongoing [6, 7]. These peptides tend to employ several mechanisms to clear pathogenic microbes. Once they interact with a pathogen, they can directly destabilize its cell membrane through pore formation, depolarization, or micellization, leading to cell lysis and leakage. They can also enter the cells and bind to or aggregate nucleic acids and protein synthesis machinery, inhibiting their normal functioning and vital metabolic processes [6, 8]. Their traits, such as level of hydrophobicity, charge distribution, and their small size, allow them to get to the targeted sites faster [9]. When several AMPs are used together in treatment, their activity is enhanced and their toxicity reduced [7].

Generally, AMPs are positively or negatively charged short peptides that possess broad‐spectrum antimicrobial activity [10]. Their nonspecific physical interaction traits with the membrane of pathogens enable them to eliminate pathogens with minimal chances of resistance development [6, 8]. As elements of the innate immune system, AMPs make up a defense mechanism that has been conserved across evolution in protecting their hosts against pathogenic microbes [11, 12]. They have remained effective throughout the course of evolution and pose less risk of selection pressure than conventional antimicrobials [11].

The AMP library keeps growing, and currently, there are over 3000 natural, 1200 synthetic, and 200 predicted AMPs, which are listed in the Antimicrobial Peptide Database (APD3) [13]. The natural AMPs with known activity have been identified from various sources, including humans (154), plants (268), mammals (397), birds (47), amphibians (1110), fish (150), reptiles (55), mollusks (54), protozoa (6), and arthropods (677), including insects (397) [13]. The microbe‐rich aquatic and moist environments of the amphibians make their porous and permeable skin highly susceptible to infections [14]. On the other hand, the open circulatory system in arthropods makes them vulnerable to the rapid spread of pathogens [15]. Due to this, there is a massive diversity of AMPs produced by these organisms [13].

In insects, AMPs are expressed either constitutively or generated in response to pathogen invasions and any other immune challenges [16, 17]. Their synthesis occurs in various tissues and cells, after which they are released into the haemocoel to eliminate pathogens [18]. These tissues and cells include fat body, hemocytes, salivary glands, and midgut epithelia [10, 16]. Depending on the insect species, hemocytes can undergo differentiation, giving rise to distinct cell types such as plasmocytes, granulocytes, oenocytoids, and spherulocytes [19]. These cell types also engage in the synthesis of AMPs [19]. Mostly, AMPs are normally produced across various stages of an insect’s life. However, a hike in their production can happen at certain developmental stages, especially during or immediately after molting [16, 20].

The extraction of AMPs from insects’ hemolymph has been achieved before using acidified solvents [21–23]. Considerable attention has been devoted to insects within the orders Diptera, Coleoptera, Lepidoptera, Isoptera, Hemiptera, and Hymenoptera. This has left the order Orthoptera as the least explored [21, 24–26]. One of the insects that belongs to the order Orthoptera is *Schistocerca gregaria* (the desert locust). It is among the serious threats to agriculture worldwide [27]. This is due to its voracious feeding habits on crops and vegetation, its ability to reproduce rapidly, and the formation of massive swarms that can fly up to 1.68 m/s [27, 28]. Though adversarial to agriculture, their abundance can be harnessed as a sustainable and inexhaustible source of AMPs.

Given this knowledge gap and the potential of desert locusts as a source of AMPs, this work focused on three aspects: firstly, the extraction of AMPs using acidified solvents from whole bodies of nonimmune‐challenged and immune‐challenged fourth‐instar desert locust nymphs; secondly, the fractionation and identification of the AMPs using mass spectrometric techniques; and thirdly, the assessment of the antimicrobial activity of the peptide extracts against clinical microbes. The findings of this work contribute greatly to the quest for alternative antimicrobials in combating AMR.

## 2. Materials and Methods

### 2.1. Rearing and Immune‐Challenging of Desert Locust Nymphs

The desert locusts, distinguished by their characteristic black and yellow coloration, were obtained and mass‐reared at *icipe* (01°13′17.824″ S, 36°53′47.571″ E; approximately 1600 m above sea level). The nymphs were housed in enclosed cages under controlled environmental conditions (temperature: 30°C–34°C; relative humidity: 50%–53%; 12 h light:12 h photoperiod). The locusts were fed on wheat bran and green vegetation, including young wheat seedlings, barley, and common weeds such as blackjack, black nightshade, wandering Jew, sow thistle, and gallant soldier, mimicking their natural diets.

Fourth‐instar nymphs were selected for AMP extraction and divided into two groups containing 50 each: Group 1, nonimmune‐challenged, and Group 2, immune‐challenged. Nymphs from Group 2 were injected with 10 μL (1 × 10^6^ CFU) of harvested bacterial cells (*Escherichia coli*, *Staphylococcus aureus*, and a mixture of both) at a concentration of 1 × 10^8^ CFU/mL, as well as sterile distilled water to induce a stress response. The injection site, located between the second and third abdominal sclerites, was disinfected with 70% ethanol before injection. Extraction procedures for Group 2 were performed 16 h post‐injection [29].

### 2.2. Peptide Extraction From Desert Locust Nymphs

Four acidified solvents (90:9:1, v/v) were used to extract AMPs from nonimmune‐challenged desert locust (*S. gregaria*) nymphs: (i) methanol (analytical grade, Merck)/distilled water/trifluoroacetic acid (TFA, Sigma‐Aldrich), (ii) isopropanol (analytical grade, Merck)/distilled water/TFA, (iii) ethanol (analytical grade, Merck)/distilled water/TFA, and (iv) butanol (analytical grade, Merck)/distilled water/TFA. The solvent that gave extracts with the highest antimicrobial activity and was safer to handle was subsequently used to extract peptides from immune‐challenged nymphs. To ensure solvent or reagent interference did not affect activity, a procedural negative control was used. This involved subjecting distilled water to the entire extraction procedure, and the final product was tested against the microorganisms.

Prior to extraction, desert locust nymphs were chilled at −80°C for 3 h to immobilize and inactivate them [30]. Nymphs were rinsed with water (distilled) to remove surface debris; their surfaces were sterilized with 70% ethanol and then washed and air‐dried on sterile paper towels. A sample of 50 g nymphs was homogenized in 150 mL of the acidified solvent containing 100 μL of protease inhibitor (Aprotinin, Sigma‐Aldrich, USA) using a Waring laboratory blender. The homogenized mixture was filtered through sterile muslin, sonicated (15 min), and centrifuged at 4000 rpm for 20 min. The clarified supernatant was dried under reduced pressure, and the residue was reconstituted in 0.1% TFA (Sigma‐Aldrich, USA). Lipids were sequentially removed using 15 mL of n‐hexane and 10 mL of ethyl acetate in a separating funnel. The hydrophilic fraction was evaporated to remove solvent residues, lyophilized, and preserved at −80°C until further use. The antimicrobial potency level of the resulting extracts was subsequently assessed.

### 2.3. Solid‐Phase Extraction (SPE) of Desert Locust Peptide Extracts

The cleanup of AMPs using SPE was carried out as described in [31–34] with slight modifications. Acidified‐ethanol extracts obtained from the nonimmune‐challenged and immune‐challenged nymphs were used in this extraction. Briefly, approximately 30 mg of lyophilized extract, representing 5% of the cartridge loading capacity, was suspended in 5 mL of an aqueous TFA (0.1% v/v; pH ≈ 2.0) to aid solubilization, followed by ultrasonication for 5 min and centrifugation at 3000 rpm for 10 min. LC‐18 SPE solid phase cartridges (45 μm particle size, 60 Å pore size, column volume 6 mL) containing 500 mg bed weight (Supelclean, Sigma‐Aldrich) were activated and equilibrated using methanol (3 mL) and 0.1% TFA (3 mL) in water (v/v). The clarified sample solution was loaded and slowly passed at a 1‐mL/min flow rate, allowing peptides to bind while hydrophilic contaminants such as salts were removed in the flow‐through and subsequently washed with 5 mL of an aqueous 0.1% TFA solution. Peptides were eluted thrice with 3 mL of 70% (v/v) acetonitrile supplemented with 0.1% TFA. Combined eluates were dried under vacuum at 30°C to remove organic solvent and residual TFA and stored at −80°C. Later, the SPE‐obtained samples (SPE A from nonchallenged nymphs and SPE B from challenged nymphs) were reconstituted to make various concentrated solutions, and their toxicity against erythrocytes and bioactivity were assessed through hemolytic and minimum inhibitory concentration (MIC) assays, respectively.

### 2.4. Fractionation and Mass Spectrometric Analysis of Peptides

Acidified‐ethanol extracts from immune‐challenged and nonimmune‐challenged nymphs were loaded onto a Sephadex LH‐20 column (Sigma‐Aldrich, Massachusetts, USA), 25 × 2.5 cm (L × D), and eluted with methanol to fractionate the components. The separated layers were collected in fractions (20 in number, containing approximately 5 mL each) at a flow rate of 5.6 μL/s, and 100 μL of the solution was transferred into HPLC vials containing inserts. Fractions were subjected to RP‐HPLC (Shimadzu, Japan) fitted with a C18 column (Agilent Zorbax Eclipse, 5.0 μm, 4.6 × 150 mm) with a flow rate of 1.0 mL/min. The samples were detected at a wavelength range of 190–210 nm, followed by identification with an Orbitrap Exploris 480 mass spectrometer (Thermo Fisher Scientific, Domierstrasse 4, 82110 Germering, Germany). Chromatographic separation was performed using a 25‐min gradient. The run started at 5% B (0–2 min, isocratic), followed by a linear rise to 40% B over 2–6 min and to 80% B by 15 min. The proportion of Solvent B was then rapidly increased to 100% between 18 and 19 min and maintained for 3 min. The column was reequilibrated to 5% within 1 min and held for an additional 2 min (23–25 min). Elution employed 0.1% formic acid in water (A) and acetonitrile (B) at a 0.2‐mL/min flow rate. Mass spectra were acquired in data‐dependent mode using a HESI source, over an m/z range of 150–2000 m/z with a resolving power of 480,000. The obtained data were processed using Thermo FreeStyle software, and peak lists were generated for further analysis.

### 2.5. Sequence Analysis, AMP Prediction, and Physicochemical Parameters

The LC‐Orbitrap‐MS spectra peak lists were analyzed against target peptide sequences contained in a FASTA file retrieved from the UniProt database (https://www.uniprot.org/), filtered for *Schistocerca* species [35]. To filter out laboratory protein contaminants, the search sequences were appended with the common Repository of Adventitious Proteins, cRAP, database (https://www.thegpm.org/crap/). Peptide identification was performed using SearchGUI Version 4.3.11 [36], employing X! Tandem software and MS‐GF + search engines. Cleavage was set to unspecific/no enzymes to target endogenously cleaved insect peptides. Carbamidomethylation of cysteine (+57.0215) and oxidation of methionine (+15.9949) were set as fixed and variable posttranslational modifications, respectively. The precursor and fragment mass tolerance were maintained at 10 ppm and 0.02 Da, respectively. Spectrum identifications were processed using PeptideShaker Version 3.0.11 [37] to infer peptides and controlled by setting the false discovery rate (FDR) to 1% at both peptide and protein levels. Peptide spectrum matches (PSMs) were then manually validated using raw spectral data (fragmentation conducted via higher‐energy collisional dissociation) on Thermo FreeStyle 1.8 SP2 QF1 software. This was performed by evaluating the matching b‐ and y‐ion fragment series against the PSM’s corresponding MS^2^ precursor ion mass at 0.02‐Da fragment mass tolerance. Theoretical mass‐to‐charge (m/z) values and chemical structures were further confirmed using ChemDraw Professional software (PerkinElmer, Version 16.0.1.4).

Homology prediction of the identified peptide sequences was achieved using the AMP database (https://aps.unmc.edu/), and physicochemical properties were determined using ProtParam [13, 38]. Antimicrobial confidence levels were evaluated using computational algorithms, including CAMPR4, PEPNet, and AMP Scanner v2 [39–41]. The analyzed peptides were then grouped into four confidence categories: high‐confidence AMPs (predicted as AMPs by all three tools), moderate‐confidence AMPs (predicted by two tools), low‐confidence AMPs (predicted by one tool), and non‐AMPs (not predicted as AMPs by any tool).

### 2.6. Test Organisms and Culture Media for the Antimicrobial Assay

The following clinical isolates and reference microorganisms were used to evaluate the in vitro antimicrobial activity of desert locust nymph peptide extracts: *E. coli. Pseudomonas aeruginosa*, *Streptococcus agalactiae*, *Bacillus subtilis*, *Staph. aureus*, *Listeria monocytogenes*, *Salmonella Typhimurium*, *Klebsiella pneumoniae*, *Candida albicans*, *Enterococcus faecalis*, *E. coli* ATCC 29922 (quality control strain), and *Staph. aureus* ATCC 25923 (quality control strain). Pure cultures of these microorganisms were obtained from the microbiology laboratory culture collection at the VPMP department, University of Nairobi. The antimicrobial susceptibility test of the test microorganisms was undertaken in reference to the Clinical and Laboratory Standards Institute (CLSI) guidelines using Oxoid antimicrobial discs [42]. The drugs tested included ampicillin (AMP), tetracycline (TE), nitrofurantoin (NIT), nalidixic acid (NA), streptomycin (S), sulfamethoxazole (SX), co‐trimoxazole (COT; sulfa/trimethoprim), and gentamicin (GEN).

For the antibacterial assays, Mueller–Hinton (MH) agar (Oxoid CM0337, Thermo Fisher Scientific, UK) and MH broth (Oxoid CM0405M, Thermo Fisher Scientific, UK) were used. Bacterial cultures were maintained on nutrient agar (Oxoid CM0003, Thermo Fisher Scientific, UK). Sabouraud’s dextrose agar (Oxoid CM0041, Thermo Fisher Scientific, UK) was used to culture the yeast, and its antimicrobial assay was carried out on MH agar (Oxoid CM0337, Thermo Fisher Scientific, UK) supplemented with methylene blue dye (0.5 μg/mL) and glucose (2%).

### 2.7. Antimicrobial Activity by Agar Well Diffusion Assay

Determination of antimicrobial potency of the peptide extracts by this method was done according to the CLSI M100 guidelines [42]. Test microorganisms were freshly cultured and suspended in sterile saline, and the concentration was adjusted to 0.5 McFarland. The microbial suspension was then uniformly spread onto MH agar plates. Wells (6 mm diameter) were then aseptically created on the agar surface, and 60 μL of each test sample was added to individual wells: peptide extracts (100 μg/mL), fluconazole (5 mg/mL; positive control yeast), TE (30 μg/mL; bacteria positive control), and 0.1% TFA (negative control). Cultured plates were incubated at 37°C for 16 h for bacterial strains and 24 h for the yeast. The zones of inhibition (ZOI) were measured after the incubation period. In this assay, eight independent biological replicates (*N* = 8) comprising four crude peptide extracts (*N* = 4) from nonimmune‐challenged nymphs and four (*N* = 4) from immune‐challenged nymphs were evaluated. Each extract was tested in technical triplicates (*n* = 3) across a panel of 9 bacteria and 1 yeast (10 clinical microbes) [43].

### 2.8. Determination of MICs

The MICs of each crude peptide extract (*N* = 8 independent biological extractions: *N* = 4 from nonimmune‐challenged nymphs and *N* = 4 from immune‐challenged nymphs) were determined using serial dilutions in sterile 96‐well U‐bottom plates (Thermo Scientific, Massachusetts, U.S.), following CLSI guidelines with slight modifications [44]. For the extracts, 100 μg/mL solution was serially diluted to obtain a concentration scale of 50, 25, 12.5, 6.25, 3.125, 1.562, 0.78, and 0.39 μg/mL. For the positive control (30 μg/mL), the dilutions were as follows: 15, 7.5, 3.75, 1.88, 0.94, 0.47, 0.24, and 0.12 μg/mL. Briefly, 100 μL of desert locust nymph peptide extracts (100 μg/mL) and 30 μg/mL TE were separately added to the first column wells of the plate. MH broth (50 μL) was then added to the 2^nd^ column wells all the way to the 12^th^ column wells, and serial dilutions were performed by micropipetting 50 μL from the first column to the second column, and so on until the 10th column. The test microorganism, diluted 1:100 in MH broth, was added (50 μL) to all wells except those in column 12. After the plates were incubated (37°C; 16 h), 20 μL of *p*‐iodonitrotetrazolium chloride (0.02% INT, Sigma‐Aldrich, Massachusetts, U.S.) was introduced to all wells and incubated for an additional 30 min. The MICs were recorded as the last well with no color change (colorless) in each row. Each extract was tested in technical triplicate (*n* = 3) across nine clinical bacteria [45]. The MICs of the solid‐phase extracted fractions (SPE A and B) were also determined using the same procedure, with each fraction (25 μg/mL) evaluated in technical replicates (*n* = 3) against six clinical bacterial strains.

### 2.9. Hemolytic Assay

Fresh whole blood was collected from a healthy sheep, and erythrocytes were isolated by centrifugation (500 × *g*, 15 min), washed with phosphate‐buffered saline (PBS) thrice, and resuspended to a final 4% (v/v) suspension. The erythrocyte suspension (100 μL) was mixed with PBS (negative control), 100 μL SPE peptide extracts dissolved in PBS, and 0.1% (v/v) Triton X‐100 in PBS (positive control). After incubation (37°C; 1 h), the three different mixtures were centrifuged at 3000 rpm for 10 min at 4°C. Supernatants were introduced to a 96‐well plate, and the measurement of absorbance (540 nm) was carried out using a microplate reader (Epoch, BioTek/Agilent). The percentage hemolysis was computed as follows:

(1) % Hemolysis=Asample plus RBC – APBS plus RBC onlyATriton x−100 plus RBC−APBS plus RBC control×100

The hemolysis assay was evaluated in technical triplicates (*n* = 3) for each independent SPE fraction (*N* = 2; SPE A and SPE B).

### 2.10. Statistical Analysis

Analysis of the antimicrobial data was executed using R Version 4.4.2 [46] and the “dplyr” package [47]. The Shapiro–Wilk test (*p* > 0.05) was employed to check how normal the data were. To assess the effect sizes of the dependent variable on the antimicrobials, a generalized linear mixed model (GLMM) with penalized quasi‐likelihood (PQL) was fitted to the right‐skewed raw data, treating microorganism and treatment as fixed effects and considering culture well plates replication as a random effect [48]. The model was fitted assuming a Gaussian distribution with a log link function. The experimental unit was defined as an individual agar well for ZOI and an individual microtiter plate well for MIC. Descriptive statistics, including mean and standard deviation, were calculated to represent the ZOI and hemolytic effects of desert locust nymph peptide extracts. To account for the logarithmic scale of dilution steps and to prevent arithmetic skewness, the MICs were reported as geometric means. These were calculated from the technical replicates (*n* = 3) of each test material. Pairwise mean comparisons were conducted with Tukey’s adjustment to identify significant differences at the 0.05 level. Data visualization was carried out using Python 3.11.6 (https://www.python.org) along with pandas [49], seaborn [50], and matplotlib [51].

## 3. Results

### 3.1. Antimicrobial Susceptibility Patterns

AST revealed distinct resistance profiles across the isolates (Table 1). *Ps. aeruginosa* was resistant to all the tested antibiotics; *L. monocytogenes* and *Ent. faecalis* were resistant to NA, and *Staph. aureus* was resistant to ampicillin. Several microorganisms, including *E. coli*, *Kl. pneumoniae*, and *Salm*. *Typhimurium*, exhibited intermediate susceptibility to multiple antibiotics, particularly ampicillin, TE, and S.

**TABLE 1:** Antimicrobial susceptibility profiles of clinical isolates.

Test organism	AMP	TE	NIT	NA	S^∗^	SX	COT	GEN
*B. subtilis*	S	S	S	S	S	S	S	S
*Ent. faecalis*	I	S	S	R	I	S	S	I
*E. coli*	I	I	S	I	I	S	S	I
*Kl. pneumoniae*	I	I	S	S	I	S	S	S
*L. monocytogenes*	S	S	S	R	S	S	S	S
*Ps. aeruginosa*	R	R	R	R	R	R	R	R
*Salm. Typhimurium*	I	S	S	I	S	S	S	S
*Staph. aureus*	R	S	S	S	S	S	S	S
*Strep. agalactiae*	I	R	S	R	S	S	S	S
*C. albicans*	na	na	na	na	na	na	na	na

*Note:* AMP, ampicillin, TE, tetracycline, NIT, nitrofurantoin, S^∗^, streptomycin, SX, sulfamethoxazole, COT, co‐trimoxazole (sulfa/trimethoprim), and GEN, gentamicin. na—test was not done. S, susceptible, R, resistant, and I, intermediate. Breakpoints interpreted according to CLSI VET01S ED6:2023 and CLSI‐MS100 ED26.

Abbreviation: NA, nalidixic acid.

### 3.2. Agar Diffusion Assay of Nymphal Peptide Extracts

Crude nymphal peptide extracts showed distinct inhibition patterns across the 10 tested microorganisms in the agar well diffusion assay, as shown in Figure 1a and Supporting Figure 1a and 1b.

**FIGURE 1:**
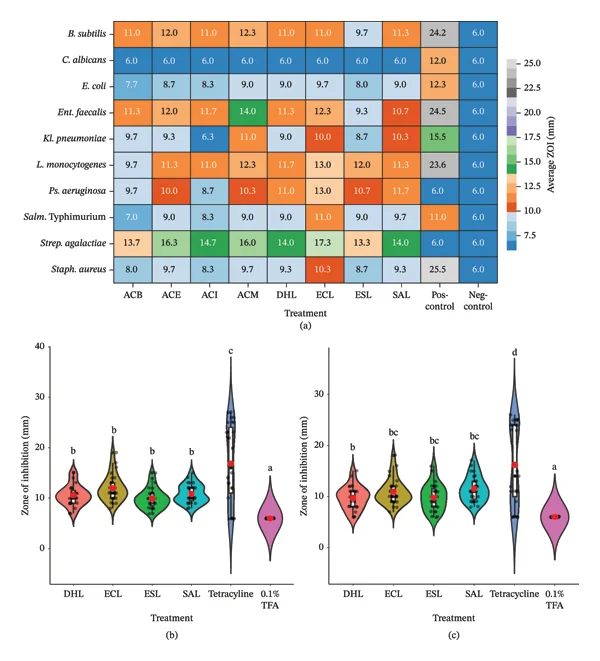
Zone of inhibition (ZOI) of nymphal peptide extracts against the tested microorganisms. (a) Average diameters of ZOI (*n* = 3) for individual extracts (*N* = 8). (b, c) Overall activity of immune (*N* = 4) and nonimmune‐challenged (*N* = 4) extracts, respectively, compared with negative and positive controls. Violin plots show distribution; red dots indicate mean values; letters denote significant differences (Tukey’s test, *α* = 0.05). Extracts from immune‐challenged nymphs: ESL (*E. coli* + *Staph. aureus*), DHL (sterile distilled water), SAL (*Staph. aureus*), and ECL (*E. coli*). Extracts from nonimmune‐challenged nymphs: ACE (acidified‐ethanol), ACI (acidified‐isopropanol), ACM (acidified‐methanol, and ACB (acidified‐butanol). Controls: positive—tetracycline (bacteria); fluconazole (yeast); negative—0.1% trifluoroacetic acid.

Within the immune‐challenged group, the ECL extract (induced with *E. coli*) produced the largest inhibition zone, 17.33 ± 1.53 mm, against *Strep. agalactiae*. While other extracts from this group also inhibited *Staph. aureus*, *L. monocytogenes*, *Ent. faecalis*, and *E. coli*, ECL consistently produced the widest inhibition zones against these pathogens (10.3 ± 1.15 mm, 13.0 ± 1.00 mm, 12.3 ± 2.31, and 9.7 ± 0.58 mm, respectively). Notably, inhibition of *Salm*. *Typhimurium* by ECL (11.0 ± 1.00 mm) was comparable to that of TE (11.7 ± 1.53 mm) as displayed in Figure 1a and detailed in Supporting Table 1.

The reference drug, TE, showed no detectable antimicrobial activity against *Ps. aeruginosa* and *Strep. agalactiae* (6.00 ± 00 mm). By contrast, all immune‐challenged extracts inhibited these pathogens, with the strongest effects recorded for extract ECL (*Ps. aeruginosa*: 13.00 ± 1.00 mm; *Strep. agalactiae*: 17.33 ± 1.53 mm). Extract SAL(*Staph. aureus* inducer) exhibited the largest inhibition zones against *Kl. pneumoniae* (10.3 ± 1.53 mm) and *B. subtilis* (11.3 ± 1.15 mm) as visualized in Figure 1a and detailed in Supporting Table 1. Across the immune‐challenged group, extract ECL showed the highest overall activity (effect size = 2.59, SE = 0.07, df = 191, *t* = 38.62) as elaborated in Supporting Table 3. None of the extracts inhibited the yeast *C. albicans,* as shown in Figure 1a and presented in Supporting Table 1.

In the nonimmune‐challenged group, the strongest activity was observed for extract ACE (acidified ethanol), which produced an inhibition zone of 16.33 ± 1.53 mm against *Strep*. *agalactiae*. Unlike TE, which showed no detectable activity against *Strep. agalactiae* and *Ps. aeruginosa* (6.00 ± 0.00 mm), all extracts in this group inhibited these pathogens, with ACE (*Strep. agalactiae*: 16.3 ± 1.53 mm; *Ps. aeruginosa*: 10.0 ± 1.00 mm) and ACM (acidified methanol; *Strep*. *agalactiae*: 16.0 ± 1.00 mm; *Ps. aeruginosa*: 10.3 ± 1.15 mm) showing the largest activity. These two extracts also exhibited the highest activity against *B. subtilis* (ACE: 12.0 ± 1.00 mm; ACM: 12.3 ± 0.58 mm), *Staph. aureus* (9.7 ± 1.53 mm each), and *Salm*. *Typhimurium* (ACE: 9.0 ± 1.00 mm each). Beyond these shared targets, ACM further outperformed other extracts against *Ent. faecalis* (14.0 ± 1.00 mm), *L. monocytogenes* (12.3 ± 1.53 mm), *Kl. pneumoniae* (11.0 ± 1.00 mm), and *E. coli* (9.0 ± 1.00 mm) as visualized in Figure 1a and elaborated in Supporting Table 2.

Collectively, three extracts from this group (ACI, ACE, and ACM) were identified as the most active extracts (Figure 1c). To distinguish their magnitude, further effect size analysis confirmed ACM as the leading extract (effect size = 2.64 ± 0.07), closely followed by ACE (effect size = 2.59 ± 0.07), with no difference between them (*p* = 0.999), as shown in Figure 1c and explained further in Supporting Tables 1 and 3. Notably, none of the extracts from this group exhibited detectable activity against the yeast *C. albicans*. Also, a significant difference (*p* ≤ 0.05) between the antimicrobial activities of nymphal peptide extracts from both groups and controls was noted (Figure 1b,c).

### 3.3. MIC of Nymphal Peptide Extracts

The crude nymphal peptide extracts exhibited a wide range of MIC values across the tested microbial panel, as shown in Figure 2a and Supporting Figure 1c.

**FIGURE 2:**
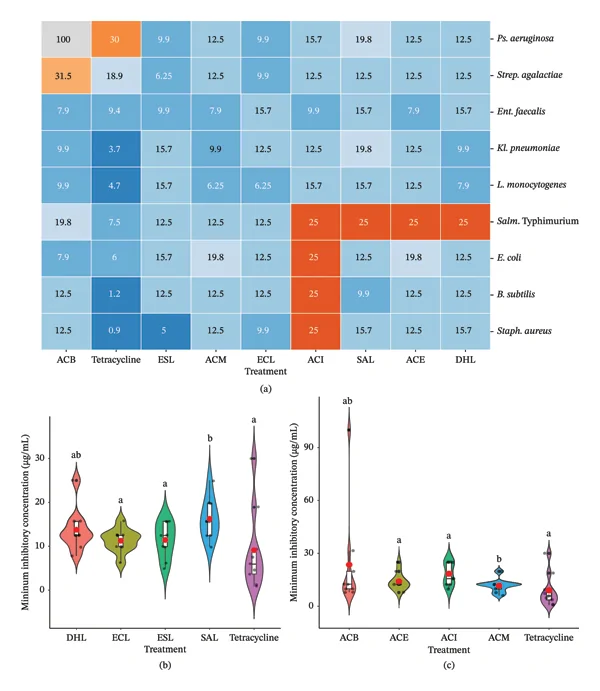
Minimum inhibitory concentration (MIC) of nymphal crude peptide extracts. (a) Geometric mean MIC values calculated across technical replicates (*n* = 3) for each independent peptide extract against the tested bacterial pathogens. (b, c) Overall geometric mean MICs of (b) immune‐challenged (*N* = 4) and (c) nonimmune‐challenged (*N* = 4) extract groups calculated across the panel of the tested bacterial pathogens, presented alongside the positive control (an assay reference standard). Violin plots indicate distribution, with mean values shown as red dots and distinct letter caps denoting significant differences (Tukey’s tests, *α* = 0.05). Extracts from immune‐challenged nymphs: ESL (*E. coli* + *Staph. aureus*), DHL (sterile distilled water), SAL (*Staph. aureus*), and ECL (*E. coli*). Extracts from nonimmune‐challenged nymphs: acidified‐ethanol (ACE), acidified‐isopropanol (ACI), acidified‐methanol (ACM), and acidified‐butanol (ACB). Control: positive—tetracycline.

MIC values of extracts from the immune‐challenged group ranged from 5 to 25 μg/mL. Lowest overall MICs were recorded for extract ESL against *Staph. aureus* (5 μg/mL) and*Strep. agalactiae* (6.25 μg/mL), and for the extract ECL against*L. monocytogenes* (6.25 μg/mL) (Figure 2a). These two extracts were particularly potent, with ranges of 5–15.7 μg/mL and 6.25–15.7 μg/mL, respectively, and both inhibited *Ps. aeruginosa* at 9.9 μg/mL, markedly lower than TE (30 μg/mL). Extracts DHL (12.5 μg/mL) and SAL (19.8 μg/mL) also exhibited lower MICs than the reference drug TE against this pathogen.

For other tested microbes, DHL, ESL, and SAL inhibited *Kl. pneumoniae*, *Ent. faecalis*, and *B. subtilis,* respectively, at 9.9 μg/mL. ECL and SAL inhibited *E. coli*, while ESL and ECL inhibited *Salm*. *Typhimurium*, both at 12.5 μg/mL. There was no significant difference between the activities of DHL, ECL, and ESL and those of TE (*p* > 0.05) (Figure 2b).

By contrast, MIC values of peptide extracts from nonimmune‐challenged nymphs ranged from 7.9 to 100 μg/mL across all tested microorganisms (Figure 2a). Extracts ACM, ACI, and ACE inhibited *Ps. aeruginosa* at 12.5, 15.7, and 12.5 μg/mL, respectively, all lower than TE (30 μg/mL). Similarly, ACB, ACM, and ACE achieved 7.9 μg/mL against *Ent. faecalis*, compared with 9.4 μg/mL for TE. Notably, ACM inhibited *L. monocytogenes* at 6.25 μg/mL, and ACB inhibited *E. coli* at 7.9 μg/mL, while both extracts achieved 9.9 μg/mL against *Kl. pneumoniae*. Extracts ACM, ACI, and ACE each achieved 12.5 μg/mL against *Strep. agalactiae*. A MIC value of 12.5 μg/mL was also observed for ACM, ACE, and ACB against *Staph. aureus*; ACE, ACM, and ACB against *B. subtilis*; and ACM against *Salm*. *Typhimurium* (Figure 2a). Within this group, no significant differences were detected among the extracts (*p* > 0.05), whereas all extracts differed significantly from TE (*p* ≤ 0.05) (Figure 2c).

Overall, extracts ACM (1.82 ± 0.32) and ACE (1.95 ± 0.30) exhibited a smaller MIC size effect, comparable to TE (2.00 ± 0.30) in the nonimmune‐challenged group. Notably, the immune‐challenged extract ECL (2.16 ± 0.17) showed a smaller MIC size effect than its nonimmune counterpart ACE (effect size = 2.37 ± 0.16), as elaborated in Supporting Table 3.

### 3.4. Hemolytic Activity of SPE‐A and SPE‐B

Peptide extracts from nonimmune‐challenged (SPE‐A) and immune‐challenged (SPE‐B) locust nymphs induced < 5% hemolysis in mammalian red blood cells across 10–200 μg/mL, remaining within the ASTM‐F756‐17 non‐ to slightly hemolytic range (Table 2, Figure 3). At 10 μg/mL, SPE‐B was nonhemolytic (1.31 ± 0.23%), whereas SPE‐A was slightly hemolytic (3.02 ± 0.12%). At 25 μg/mL, both extracts showed slight hemolysis (SPE‐B: 2.37 ± 0.20%; SPE‐A: 3.29 ± 0.11%), with significant group differences at both concentrations (*p* < 0.001 and *p* = 0.002, respectively). At higher concentrations (50–200 μg/mL), hemolysis ranged from 3.35% to 4.21%, with SPE‐B lower than SPE‐A.

**TABLE 2:** Hemolysis of peptide extracts SPE‐A (nonimmune) and SPE‐B (immune) in mammalian red blood cells across different concentrations.

Sample	10 μg/mL	25 μg/mL	50 μg/mL	100 μg/mL	200 μg/mL
SPE‐A	3.02 ± 0.12	3.29 ± 0.11	3.48 ± 0.12	3.81 ± 0.10	4.21 ± 0.31
SPE‐B	1.31 ± 0.23	2.37 ± 0.20	3.35 ± 0.20	3.68 ± 0.12	4.01 ± 0.30

*Note:* Values are expressed as mean ± SD.

**FIGURE 3:**
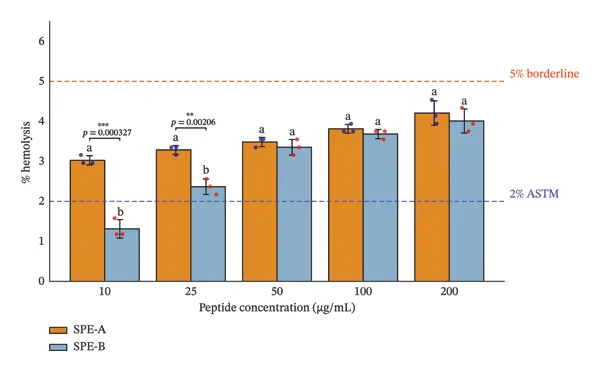
Hemolytic activity of peptide extracts SPE‐A (nonimmune) and SPE‐B (immune) in mammalian red blood cells. Distinct letters denote significant mean differences (Tukey’s tests, *α* = 0.05). Hemolysis values are compared with thresholds defined by the American Society for Testing and Materials (ASTM‐F756‐17): < 2% nonhemolytic, 2%–5% slightly hemolytic, and > 5% hemolytic.

### 3.5. MIC of SPE‐A and SPE‐B

Both SPE‐A and SPE‐B exhibited MIC values greater than 25 μg/mL against all six tested pathogens (*Staph. aureus, Salm. Typhimurium*, and *Strep. agalactiae, E. coli, Ent. faecalis, and Ps. aeruginosa*) (Figure 4). In contrast, TE inhibited *Staph. aureus* at 0.47 μg/mL and *Salm*. *Typhimurium* at 0.94 μg/mL, while higher MICs were observed for *E. coli and Ent. faecalis* (> 30 μg/mL) and for *Ps*. *aeruginosa* and *Strep. agalactiae* (30 μg/mL). Overall, no measurable MICs within the tested concentration range (≤ 25 μg/mL) were detected for the peptide extracts.

**FIGURE 4:**
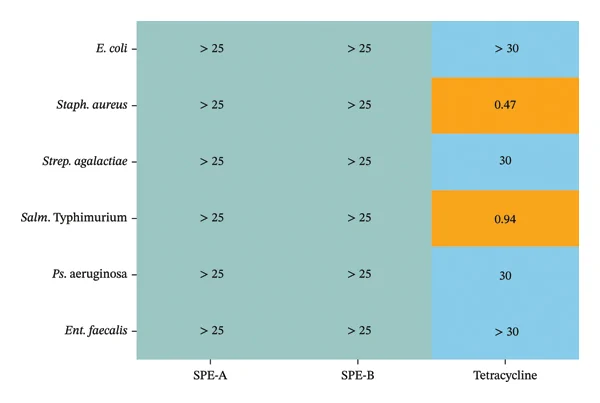
Minimum inhibitory concentrations (MICs, μg/mL) of SPE‐A and SPE‐B against six bacterial isolates. The sign “>” indicates no inhibition at the maximum concentration tested (25 μg/mL for extracts and 30 μg/mL for tetracycline).

### 3.6. Fractionation and Identification of Desert Locust Nymph Peptides

From the analysis of the obtained MS/MS data, a total of 96 unique cationic peptide sequences were identified (Figure 5). Among them, 84 were exclusively present in immune‐challenged nymphs, while 9 were unique to nonimmune‐challenged nymphs. Interestingly, 3 peptide sequences (ATKAK, ASGAIK, and AKANA) were shared between the two groups. In total, 87 peptides were detected in the immune‐challenged nymphs, compared to only 12 in the nonimmune‐challenged nymphs (Figure 5). The identified peptides had observed mass values ranging from 474.26 to 3615.78 Da, with sequence lengths varying between 5 and 30 amino acids. Notably, most of the sequences consisted of 5–12 amino acids, as indicated in Supporting Tables 4 and 5.

**FIGURE 5:**
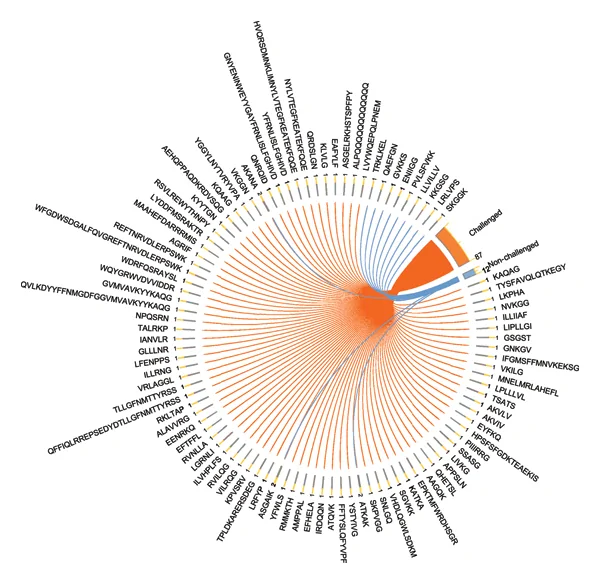
Peptide sequences from immune‐challenged and nonimmune‐challenged desert locust. Total peptide sequences detected in immune‐challenged and nonimmune‐challenged desert locust nymphs.

### 3.7. Peptide Sequence Analysis and AMP Prediction

Among the 96 unique peptide sequences analyzed using three independent AMP prediction tools, 11 were classified as high‐confidence AMPs, 26 as moderate‐confidence AMPs, 31 as low‐confidence AMPs, and 28 as non‐AMPs (Figure 6). The PepNet tool predicted a total of 42 peptides as AMP candidates, while CAMPR4 and AMP Scanner v2 predicted a total of 39 and 36 peptides, respectively (Figure 7a).

**FIGURE 6:**
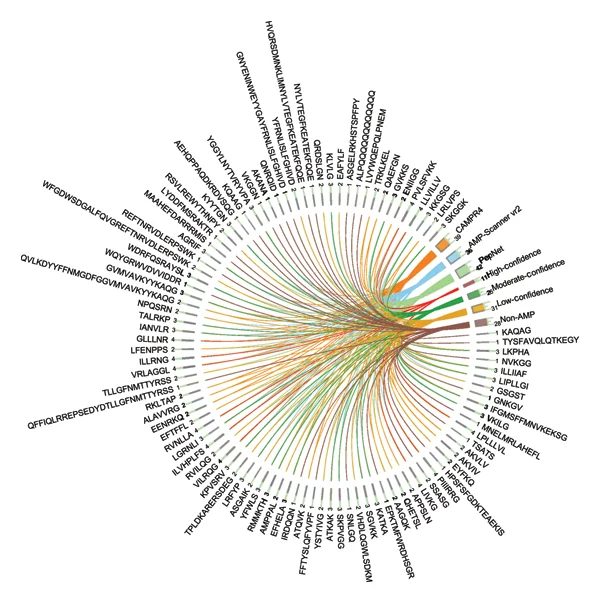
Prediction of desert locust peptide sequence groups using AMP prediction tools. Three independent computational tools were employed in prediction: CAMPR4, AMP Scanner vr2, and PepNet.

**FIGURE 7:**
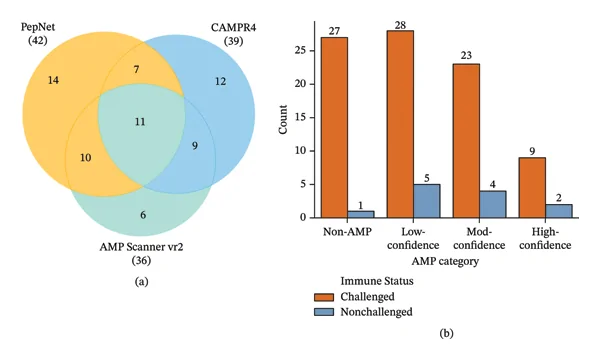
Peptide prediction tools’ agreement and prediction categories. (a) Performance and agreement among the three independent AMP computational tools. (b) Number of the identified peptides in each group (challenged and nonchallenged) categorized by confidence level (mod‐confidence—moderate confidence). Three peptides, categorized as mod‐confidence, low‐confidence, and non‐AMP, were detected in both groups (challenged and unchallenged).

From the 11 high‐confidence predicted AMPs, 9 were identified in the immune‐challenged group and 2 in the nonimmune‐challenged group. For moderate‐confidence predictions, 23 peptides were recorded in the immune‐challenged group and 4 in the nonimmune‐challenged group. Low‐confidence predicted AMPs were predominantly from the immune‐challenged group (28 peptides) compared to 5 from the nonimmune‐challenged group. Finally, 27 non‐AMP peptides were identified in the immune‐challenged group, while 1 was observed in the nonimmune‐challenged group (Figure 7b).

### 3.8. Peptide Homology Analysis and Physicochemical Characterization

The homology analysis of high‐ and moderate‐confidence AMP sequences revealed a similarity range of 33.33%–66.67% with known AMPs (Figure 8). The peptide sequence ILLRNG shared similarity with Desotamide A (a cyclic hexapeptide), while LLVILLV exhibited 62.5% similarity with Gageostatin A (a linear lipopeptide). The activities of the closest known AMPs included antibacterial (targeting both Gram‐positive and Gram‐negative bacteria), anti‐Gram‐positive, anti‐Gram‐negative, or antibacterial‐antifungal properties as presented in Figure 8 and Supporting Tables 4 and 5.

**FIGURE 8:**
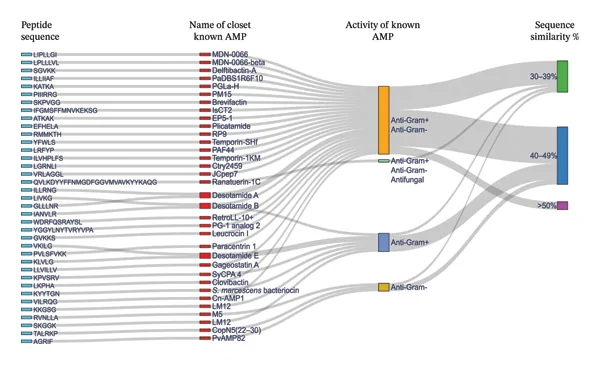
Peptide homology analysis. High‐ and moderate‐confidence AMP sequences alongside their closest known AMPs. The sequence similarity percentage (% Sim.) and the activity of the known AMPs are also shown.

The isoelectric point (pI), hydrophobic ratio, and electric charge of high‐ and moderate‐confidence AMPs ranged from 5.52 to 12.00, 0% to 100%, and +1 to +2, respectively. Notably, sequence PIIIRRG exhibited the highest pI (12.00), while sequence ILLIIAF displayed the highest hydrophobic ratio (100%) (Figure 9).

**FIGURE 9:**
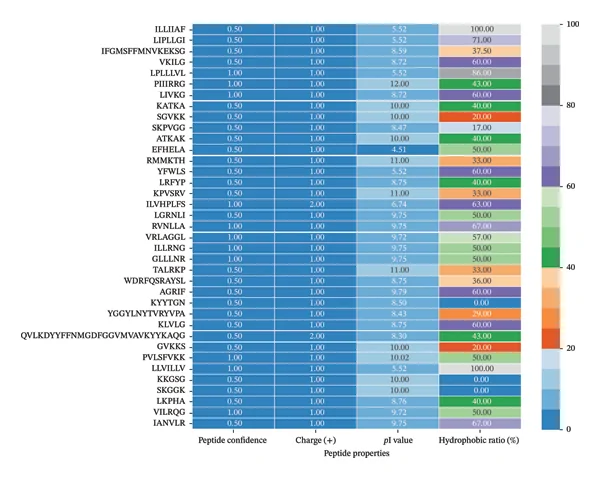
The physicochemical properties of the high‐ and moderate‐confidence AMP sequences. Peptide confidence 0.50 represents the moderate‐confidence AMPs, while 1.00 peptide confidence represents high‐confidence AMPs.

## 4. Discussion

The broad‐spectrum activity of AMPs against most pathogens makes them ideal candidates for exploration in search of new antimicrobials [26]. In this study, therefore, we focused on the extraction of peptide‐containing extracts from both immune‐challenged and nonimmune‐challenged desert locust nymphs. We further assessed their activity against clinical pathogens and identified the peptides using LC‐Orbitrap‐MS.

Acidified solvents have been recommended for extracting peptides from biological samples. They effectively recover the AMPs by denaturing proteases, dissolving basic peptides, preventing peptide degradation, and precipitating unwanted large proteins [21, 23]. This supports the successful extraction of peptides using various acidified solvents from the desert locust in the current study.

The extracted peptides showed varying antibacterial activity against both Gram‐positive and Gram‐negative bacteria, aligning with previous studies that have reported activity of insect extracts against bacteria [24, 29, 52]. The lower MICs for crude (9.9–19.8 μg/mL) and SPE (> 25 μg/mL) peptide extracts observed against the multidrug‐resistant *Ps. aeruginosa* suggest the presence of highly potent compounds that warrant further purification. This observation aligns with the previous findings that showed activity of peptide fractions against *Ps. aeruginosa* with MICs of 93–187 μg/mL [53]. *Ps. aeruginosa* is one of the WHO priority pathogens, and it is intrinsically resistant to most antibiotics, making its infections hard to treat and thus necessitating the search for workable treatment against it [4].

Despite the cleaning up of the extracts using the SPE method, these fractions (SPE A and SPE B) showed higher MIC values compared to the MICs from the crude extracts. This can be attributed to the disruption of the natural synergistic interactions between the peptides found in the crude matrix [7, 54]. Moreover, hydrophobic peptides tend to bind nonspecifically and irreversibly to the solid‐phase matrix, which can then lead to potential minor yield loss during the fractionating process [31, 32].

An enhanced activity of extracts from nymphs challenged using *E. coli* (12.5 μg/mL) was also noted, compared to extracts from the unchallenged nymphs (19.8 μg/mL), against *E. coli*. This trend remained true for all the other tested bacterial pathogens except for *Ent. faecalis*. This observation corroborates the findings of Giglio et al. [53], where peptide fractions from insects infected with *E. coli* showed an increased antimicrobial activity against *E. coli* (from 46 μg/mL to 23 μg/mL). This suggests that immunologically challenging some insects with certain pathogens increases production of immune peptides, enhancing the bioactivity of extracts obtained from them.

The MICs of several peptide extracts were lower than the MIC of the reference drug TE (30 μg/mL) across the tested pathogens. As a standard and a purified antibiotic compound, TE was employed as a positive control and a benchmark to validate the antimicrobial assays [42]. It possesses different composition and pharmacodynamic metrics compared to crude extracts, which are made up of complex mixtures of bioactive compounds [20]. The compounds in the extracts may display a synergistic effect, enhancing their potency [55]. Therefore, their comparison serves as a benchmark of biological relevance rather than a direct potency competitor.

No extract activity was noted against the yeast *C. albicans* in the current study. This may be due to structural differences between *C. albicans* and bacteria, affecting their susceptibility level. The presence of efflux pumps, affinity alteration potential, and polymorphic nature in *C. albicans* allows it to quickly develop resistance against most antimicrobials [56–59]. Therefore, the ineffectiveness of the extracts against *C. albicans* could stem from the complex structure and resistance mechanisms of this pathogen, or the insufficient potency of the peptide extracts against it.

Currently, no standardized safety threshold exists for AMPs, though hemolysis below 10% in vitro is commonly interpreted as low hemotoxicity [60–62]. According to ASTM‐F756‐17, which classifies hemolysis as hemolytic (> 5%), slightly hemolytic (2%–5%), and nonhemolytic (< 2%), the tested peptide extracts from desert locust nymphs fall within the non‐ to slightly hemolytic range [63]. While these results demonstrate low hemolytic activity, the conclusion of minimal toxicity is limited to the tested animal red blood cells. Therefore, assessing hemolysis with human erythrocytes, which are generally more sensitive than animal cells, will provide a more comprehensive evaluation of the safety of these peptide extracts [60].

Interestingly, more peptides were detected in immune‐challenged nymphs (84) compared to nonimmune‐challenged ones (9). Moreover, the majority of the peptides predicted as high and moderate confidence were from the immune‐challenged group. This finding aligns with the previous studies and also lends weight to the hypothesis that immune challenges stimulate production of AMPs in insects [64]. The three peptide sequences (ATKAK, ASGAIK, and AKANA) found in both nymph groups are evidence of constitutively expressed peptides [11].

The sequence similarity (33.33%–66.67%) observed between peptides found in the current study and those in the ADP database shows their relatedness to the reported ones. These peptides may possess similar or even superior antimicrobial activity to their closest relatives in the database. However, small differences in their sequences could dictate their structural stability and mode of action [65]. This corroborates a previous finding that reported a peptide derived from spider venom, which showed a similar activity and sequence similarity (44%) to AMP melittin from honeybee venom [38].

The sequence length (5–30 amino acids) and the charge (+1 to +2) of the identified peptides align closely with those of natural AMPs. By definition, AMPs are cationic, short‐chain peptides that are likely to disrupt pathogen membranes by interacting with their negatively charged surfaces through electrostatic interactions [8]. The short length of these peptides makes them cost‐effective to produce, a great advantage over longer AMPs [8]. Moreover, the variation in the physicochemical properties of the identified peptides suggests that each one could be tailored to target one or more bacterial strains [7].

The hydrophobicity (0%–100%) of the identified peptides positions them as potential candidates that can thwart bacterial growth and also form effective synergistic pairs [7]. An increase in peptide hydrophobicity has been shown to boost its antimicrobial activity. Furthermore, peptides that form synergistic pairs often have differing hydrophobicity, and the larger the difference, the lower the chances of pathogens acquiring resistance against them [7, 9].

While the precise modes of action of the peptide extracts in this study were not empirically determined, the physicochemical traits of the identified peptides mirror those of AMPs. The cationic nature of the identified peptides is vital in interacting with the negatively charged regions of the bacterial cell wall [60]. The hydrophobicity levels of these peptides also enhance interaction with the hydrophobic lipid core of the bacterial membrane [7]. We therefore hypothesize that the peptide extracts in this study may have acted through the disruption of the bacterial cell membrane and the integrity of the lipid bilayer, leading to cell lysis. The varying hydrophobicity levels observed among the identified peptides could lead to the formation of synergistic pairs, immune to bacterial resistance, and could act on MDR pathogens, including *Ps. aeruginosa* [7].

## 5. Conclusion

This preclinical and exploratory study highlights the potential of peptides derived from desert locust nymphs as antimicrobial agents against bacterial pathogens. The bioactivity exhibited by the crude and semipurified peptide extracts against clinical bacterial isolates, including multidrug‐resistant strains, necessitates their further investigation. In this work, immune‐challenged desert locust nymphs have been shown to increase the number of bioactive peptides produced by the nymphs, increasing the chances of finding novel molecules with broad bioactivity. The identification of the peptides from these nymphs expands the library of insect‐derived peptides with a potential therapeutic role. Although some peptides were classified as low‐confidence or non‐AMPs by computational predictions, experimental validation is required before dismissing their potential or to better understand their primary function. For peptides with higher confidence scores, more studies on their purification, stability, toxicity, and in vivo efficacy are required. Overall, our findings provide a foundation for further studies aimed at the purification, peptide synthesis, functional validation, pharmacological action, and in vivo testing of the characterized peptides and developing them into the next generation of antimicrobial agents.

## Author Contributions

Peter M. Mwangi: conceptualization, methodology, investigation, validation, data collection and analysis, visualization, original manuscript drafting, and review and editing. Chrysantus M. Tanga: investigation, methodology, visualization, resources, fund acquisition, validation, conceptualization, supervision, review and editing, and project superintendence. Lilly C. Bebora, Xavier Cheseto, and James K. Gathumbi: supervision, conceptualization, methodology, and review and editing. Cynthia M. Mudalungu and Segenet Kelemu: conceptualization, fund acquisition, validation, and review and editing. All authors reviewed the manuscript.

## Funding

The authors thank the Norwegian Agency for Development Cooperation, the Europe Commission (NESTLER Project: 101060762 and INNOECOFOOD Project: 101136739), the Australian Centre for International Agricultural Research (ACIAR) (ProteinAfrica—Grant No: LS/2020/154), the Swedish International Development Cooperation Agency (Sida), the Curt Bergfors Foundation Food Planet Prize Award, Bill & Melinda Gates Foundation (INV‐032416), the Swiss Agency for Development and Cooperation (SDC), the German Federal Ministry for Economic Cooperation and Development (BMZ), the Government of Norway, and the Government of the Republic of Kenya for their financial support.

## Disclosure

All authors approved the manuscript submission. Funders were not involved in the planning, conducting, or reporting of this study. All views presented in this manuscript are those of the authors and do not necessarily reflect the official opinion of the donors.

## Ethics Statement

Ethical clearance for this work was granted by the Biosecurity, Animal Use, and Ethics Committee, Faculty of Veterinary Medicine, University of Nairobi (REF: FVM BAUEC/2022/338). All procedures involving animals were conducted in compliance with institutional and national guidelines.

## Conflicts of Interest

The authors declare no conflicts of interest.

## Supporting Information

Additional supporting information can be found online in the Supporting Information section.

## Supporting information


**Supporting Information** Supporting Table 1: Zones of inhibition (in millimeters) of immune‐challenged desert locust nymph extracts. Supporting Table 2: Zones of inhibition (in millimeters) of nonimmune‐challenged desert locust nymph extracts. Supporting Table 3: GLMMPQL effect sizes of treatments on zone of inhibition and minimum inhibitory *concentration*. Supporting Table 4: Peptides from nonimmune‐challenged desert locust. Supporting Table 5: Peptides from immune‐challenged desert locust nymphs. Supporting Figure 1: Representative images of the antimicrobial activity assays. (a) Zones of inhibition exhibited by immune‐challenged desert locust peptide extracts and controls against *Klebsiella pneumoniae*. A—0.1% trifluoroacetic acid (negative control), B—tetracycline (positive control), C—extract with *Escherichia coli* + *Staphylococcus aureus* as immune inducers, D—extract with *Staph. aureus* as immune inducer, E—extract with *E. coli* as immune inducer, and F—extract with distilled water as immune inducer. (b) Zones of inhibition exhibited by nonimmune‐challenged desert locust peptide extracts and controls against *Bacillus subtilis*. A—acidified isopropanol extract, B—acidified methanol extract, C—acidified ethanol extract, D—acidified butanol extract, E—tetracycline (positive control), and F—0.1% trifluoroacetic acid (negative control). (c) A 96‐well plate minimum inhibitory concentration (MIC) assay of *Enterococcus faecalis* against desert locust extracts and a positive control (tetracycline). The wells from column 1 to 10 contained bacterial inoculum, test extract/positive control, broth, and the indicator dye iodonitrotetrazolium (INT). Wells with a uniform red color indicated concentrations that did not inhibit bacterial growth, as the presence of bacteria degraded INT into red formazan dye. The third column represents the MICs of the extracts. Wells in the 11th column acted as a growth control, containing broth with the bacterial inoculum and indicator dye, but no test extract/positive control. The wells in the 12th column were used as a sterility control, containing broth only. ACM—acidified methanol extract, ACI—acidified isopropanol extract, ACE—acidified ethanol extract, ECL—extract with *Escherichia coli* as immune inducer, SAL—extract with *Staph. aureus* as immune inducer, ESL—extract with *E. coli* + *Staph. aureus* as the immune inducer, and DHL—extract with distilled water as the immune inducer.

## Data Availability

Data are available on request from the authors.
