# Supplementary material for: In Silico Characterization of Desert Locust–Derived Antimicrobial Peptides Against Drug‐Resistant Bacteria
Source: Scientifica (Cairo). 2026 Oct 28;2026:7657843. doi: 10.1155/sci5/7657843 (PMC13715060; doi:10.1155/sci5/7657843)
Supplement: Supplementary file 1 [file SCI5-2026-7657843-s001.docx]

***In silico* Characterization of Desert Locust-Derived Antimicrobial Peptides against Drug-Resistant Bacteria**

Peter M. Mwangi^1, 2^, Lilly C. Bebora^2^, James K. Gathumbi^2^, Xavier Cheseto^1^, Cynthia M. Mudalungu^1,3^, Segenet Kelemu^1^, and Chrysantus M. Tanga^1*^

^1^International Centre of Insect Physiology and Ecology (*icipe*), Nairobi, Kenya

^2^Department of Veterinary Pathology, Microbiology and Parasitology, University of Nairobi, Nairobi, Kenya

^3^Department of Chemistry and Materials Science, The Technical University of Kenya (TUK), P.O Box 52428 - 00100, Nairobi – Kenya

**Supplementary Material**

**Supplementary Table 1 |** *Zones of Inhibition (in millimeters) of Immune-Challenged Desert Locust Nymph Extracts*

| **Microorganism** | **Treatment** | | | | | | **Chi-square**  **(χ²)** | **df** | **p-value** |
| --- | --- | --- | --- | --- | --- | --- | --- | --- | --- |
|  | **DHL** | **ECL** | **ESL** | **SAL** | **Tetracycline / Fluconazole** | **0.1%TFA** |  |  |  |
| *B. subtilis* | 11.0±1.00^b^ | 11.0±2.65^b^ | 9.7±1.53^ab^ | 11.3±1.15^b^ | 23.7±1.15^c^ | 6.0±0.00^a^ | 9340.9 | 5 | *** |
| *C. albicans* | 6.0±0.00^a^ | 6.0±0.00^a^ | 6.0±0.00^a^ | 6.0±0.00^a^ | 13.0±2.00^b^ | 6.0±0.00^a^ | 4365.8 | 5 | *** |
| *Ent. faecalis* | 11.3±2.52^b^ | 12.3±2.31^b^ | 9.3±0.58^ab^ | 10.7±2.08^b^ | 25.3±0.58^c^ | 6.0±0.00^a^ | 8069.4 | 5 | *** |
| *E. coli* | 9.0±1.00^b^ | 9.7±0.58^b^ | 8.0±1.00^ab^ | 9.0±1.00^b^ | 13.3±1.53^c^ | 6.0±0.00^a^ | 7024.9 | 5 | *** |
| *Kl. pneumonia* | 9.0±2.00^a^ | 10.0±1.00^a^ | 8.7±1.15^a^ | 10.3±1.53^a^ | 16.7±3.06^b^ | 6.0±0.00^a^ | 3084.7 | 5 | *** |
| *L.monocytogenes* | 11.7±1.15^b^ | 13.0±1.00^b^ | 12.0±2.00^b^ | 11.3±2.08^b^ | 22.3±0.58^c^ | 6.0±0.00^a^ | 7740.2 | 5 | *** |
| *Ps. aeruginosa* | 11.0±1.00^bc^ | 13.0±1.00^d^ | 10.7±0.58^b^ | 11.7±0.58^c^ | 6.0±0.00^a^ | 6.0±0.00^a^ | 8589.2 | 5 | *** |
| *Salm. typhimurium* | 9.0±1.73^b^ | 11.0±1.00^b^ | 9.0±1.00^b^ | 9.7±0.58^b^ | 11.7±1.53^b^ | 6.0±0.00^a^ | 4221.8 | 5 | *** |
| *Staph. aureus* | 9.3±0.58^b^ | 10.3±1.15^b^ | 8.7±1.53^ab^ | 9.3±1.15^b^ | 26.7±0.58^c^ | 6.0±0.00^a^ | 34128 | 5 | *** |
| *Strep. agalactiae* | 14.0±1.73^b^ | 17.3±1.53^c^ | 13.3±1.53^b^ | 14.0±1.00^b^ | 6.0±0.00^a^ | 6.0±0.00^a^ | 10097 | 5 | *** |

Zones of inhibition (ZOI) represented as mean ± standard deviation in millimeters. Means across the rows with the same letter do not differ significantly (post hoc comparisons, adjusted using the Tukey method, *p ≤* 0.05). The ZOI measurements were obtained using the agar well diffusion method, with each value denoting the average of three independent replicates. The value 6.00±0.00 was equivalent to the diameter of the well. Treatment’s p-values, degree of freedom (df), and chi-square Analysis of deviance values are shown. Significances. codes: 0 ‘***’ 0.001 ‘**’ 0.01 ‘*’ 0.05 ‘▪’ 0.1 ‘▬’ 1.

**Supplementary Figure 1 |** Representative images of the antimicrobial activity assays. (a) Zones of inhibition exhibited by immune-challenged desert locust peptide extracts and controls against Klebsiella pneumoniae. A- 0.1% trifluoroacetic acid (negative control), B- tetracycline (positive control), C- extract with Escherichia coli + Staphylococcus aureus as immune-inducers, D- extract with Staph. aureus as immune inducer, E- extract with E. coli as immune inducer, F- extract with distilled water as immune inducer. (b) Zones of inhibition exhibited by non-immune challenged desert locust peptide extracts and controls against Bacillus subtilis. A- acidified isopropanol extract, B- acidified methanol extract, C- acidified ethanol extract, D- acidified butanol extracts, E- tetracycline (positive control), F- 0.1% trifluoroacetic acid (negative control). (C) A 96-well plate minimum inhibitory concentration (MIC) assay of Enterococcus faecalis against desert locust extracts and a positive control (tetracycline). The wells from column 1 to 10 contained bacteria inoculum, test extract/positive control, broth, and the indicator dye iodonitrotetrazolium (INT). Wells with a uniform red color indicated concentrations that did not inhibit bacteria growth, as the presence of bacteria degraded INT into red formazan dye. The third column represents the MICs of the extracts. Wells in the 11th column acted as a growth control, containing broth with the bacteria inoculum, indicator dye, but no test extract/positive control. The wells in the 12th column were used as a sterility control, containing broth only. ACM - acidified methanol extract, ACI - acidified isopropanol extract, ACE - acidified ethanol extract, ECL - extract with Escherichia coli as immune inducer SAL- extract with Staph. aureus as immune inducer, ESL - extract with E. coli + Staph. aureus as immune-inducers, DHL- extract with distilled water as immune inducer.

**Supplementary Table 2 |** *Zones of Inhibition (in millimeters) of Non-Immune-Challenged Desert Locust Nymph Extracts*

| **Microorganism** | **Treatment** | | | | | | **Chi-square**  **(χ²)** | **df** | **p-value** |
| --- | --- | --- | --- | --- | --- | --- | --- | --- | --- |
|  | **ACB** | **ACE** | **ACI** | **ACM** | **Tetracycline/ Fluconazole** | **0.1% TFA** |  |  |  |
| *B. subtilis* | 11.0±1.00^b^ | 12.0±1.00^b^ | 11.0±1.00^b^ | 12.3±0.58^b^ | 24.7±0.58^c^ | 6.0±0.00^a^ | 1281 | 5 | *** |
| *C. albicans* | 6.0±0.00^a^ | 6.0±0.00^a^ | 6.0±0.00^a^ | 6.0±0.00^a^ | 11.0±1.00^b^ | 6.0±0.00^a^ | 14967 | 5 | *** |
| *Ent. faecalis* | 11.3±0.58^b^ | 12.0±2.00^bc^ | 11.7±0.58^bc^ | 14.0±1.00^c^ | 23.7±0.58^d^ | 6.0±0.00^a^ | 23844 | 5 | *** |
| *E. coli* | 7.7±1.53^ab^ | 8.7±1.53^abc^ | 8.3±1.15^ab^ | 9.0±1.00^bc^ | 11.3±0.58^c^ | 6.0±0.00^a^ | 3268.4 | 5 | *** |
| *Kl. pneumonia* | 9.7±1.53^b^ | 9.3±0.58^b^ | 6.3±0.58^a^ | 11.0±1.00^b^ | 14.3±0.58^c^ | 6.0±0.00^a^ | 20686 | 5 | *** |
| *L. monocytogenes* | 9.7±0.58^b^ | 11.3±1.15^b^ | 11.0±1.0^b^ | 12.3±1.53^b^ | 25.0±1.00^c^ | 6.0±0.00^a^ | 29595 | 5 | *** |
| *Ps. aeruginosa* | 9.7±1.53^b^ | 10.0±1.00^b^ | 8.7±2.08^ab^ | 10.3±1.15^b^ | 6.0±0.00^a^ | 6.0±0.00^a^ | 3547.8 | 5 | *** |
| *Salm.* Typhimurium | 7.0±1.00^ab^ | 9.0±1.00^cd^ | 8.3±0.58^bc^ | 9.0±0.00^cd^ | 10.3±1.15^d^ | 6.0±0.00^a^ | 5231.6 | 5 | *** |
| *Staph. aureus* | 8.0±2.00^a^ | 9.7±1.53^a^ | 8.3±2.08^a^ | 9.7±1.53^a^ | 24.3±1.15^b^ | 6.0±0.00^a^ | 10059 | 5 | *** |
| *Strep. agalactiae* | 13.7±1.53^b^ | 16.3±1.53^c^ | 14.7±1.53^bc^ | 16.0±1.00^c^ | 6.0±0.00^a^ | 6.0±0.00^a^ | 6185.9 | 5 | *** |

Zones of inhibition represented as mean ± standard deviation in millimeters. Means across the rows with the same letter do not differ significantly (post hoc comparisons, adjusted using the Tukey method, *p ≤* 0.05). The ZOI measurements were obtained using the agar well diffusion method, with each value representing the average of three independent replicates. The value 6.00±0.00 was equivalent to the diameter of the well. Treatment’ p-values, degree of freedom (df), and chi-square Analysis of Deviance values are shown. Significances. codes: 0 ‘***’ 0.001 ‘**’ 0.01 ‘*’ 0.05 ‘▪’ 0.1 ‘▬’ 1. ACB- acidified butanol extract, ACE- acidified ethanol extract, ACI- acidified isopropanol extract, ACM-acidified methanol extract, TFA- trifluoroacetic acid.

**Supplementary Table 3 |** GLMMPQL *effect sizes of treatments on zone of inhibition and minimum inhibitory concentration.*

| **Locust Nymphs Immune Status** | **Zones of Inhibition (ZOIs)** | | | | | | **Minimum Inhibitory Concentration (MICs)** | | | | | |
| --- | --- | --- | --- | --- | --- | --- | --- | --- | --- | --- | --- | --- |
|  | **Fixed Effects** | **Value** | **Std. error** | **DF** | **t-value** | **p-value** | **Fixed Effects** | **Value** | **Std. Error** | **DF** | **t-value** | **p-value** |
| Non-  immune  challenged | TreatmentACB | 2.478 | 0.073 | 162 | 34.039 | 0.00 | TreatmentACB | 3.046 | 0.248 | 120 | 12.291 | 0.00 |
|  | TreatmentACE | 2.589 | 0.068 | 162 | 37.929 | 0.00 | TreatmentACE | 1.949 | 0.300 | 120 | 6.496 | 0.00 |
|  | TreatmentACI | 2.493 | 0.072 | 162 | 34.522 | 0.00 | TreatmentACI | 2.245 | 0.275 | 120 | 8.165 | 0.00 |
|  | TreatmentACM | 2.643 | 0.066 | 162 | 39.876 | 0.00 | TreatmentACM | 1.816 | 0.316 | 120 | 5.747 | 0.00 |
|  | TreatmentFluconazole | 3.109 | 0.207 | 162 | 15.013 | 0.00 | TreatmentTetracycline | 1.997 | 0.295 | 120 | 6.768 | 0.00 |
|  | TreatmentTetracycline | 3.046 | 0.056 | 162 | 54.409 | 0.00 |  |  |  |  |  |  |
|  | TreatmentTFA | 1.998 | 0.102 | 162 | 19.512 | 0.00 |  |  |  |  |  |  |
| Immune-  challenged | TreatmentACE | 2.513 | 0.069 | 191 | 35.906 | 0.00 | TreatmentACE | 2.365 | 0.163 | 146 | 14.549 | 0.00 |
|  | TreatmentDHL | 4.778 | 0.071 | 191 | 34.727 | 0.00 | TreatmentDHL | 2.374 | 0.162 | 146 | 14.649 | 0.00 |
|  | TreatmentECL | 2.592 | 0.067 | 191 | 38.616 | 0.00 | TreatmentECL | 2.160 | 0.173 | 146 | 12.471 | 0.00 |
|  | TreatmentESL | 2.412 | 0.074 | 191 | 32.552 | 0.00 | TreatmentESL | 2.324 | 0.164 | 146 | 14.139 | 0.00 |
|  | TreatmentFluconazole | 2.230 | 0.187 | 191 | 17.284 | 0.00 | TreatmentSAL | 2.645 | 0.153 | 146 | 17.314 | 0.00 |
|  | TreatmentSAL | 2.493 | 0.071 | 191 | 35.236 | 0.00 | TreatmentTetracycline | 1.980 | 0.186 | 146 | 10.635 | 0.00 |
|  | TreatmentTetracycline | 2.980 | 0.057 | 191 | 51.999 | 0.00 |  |  |  |  |  |  |
|  | TreatmentTFA | 1.926 | 0.104 | 191 | 18.459 | 0.00 |  |  |  |  |  |  |

The effect sizes of the predictor variables, treatment, on the response variables, ZOI Diameter and MIC. The effect sizes are represented as estimated effect size values with their corresponding standard errors, t-values, degrees of freedom, and p-values. ACB-acidified butanol extracts, ACE-acidified ethanol extracts, ACI- acidified isopropanol extracts, ACM- acidified methanol extracts, TFA- 0.1% Trifluoroacetic acid. Immune-inducers acronyms: DH (distilled water), EC *(E. coli),* SA *(S. aureus),* ES *(E. coli+ S. aureus).* The last letter in these extracts denotes the term “locust”.

| **Supplementary Table 4 \|** *Peptides from non-immune-challenged desert locust* | | | | | | | | | | | | | |
| --- | --- | --- | --- | --- | --- | --- | --- | --- | --- | --- | --- | --- | --- |
| Peptide Sequence | Fraction | Retention  Time | Charge | Observed  m/z | Theoretical m/z | Δppm  error | PI Value | Aliphatic  Index | Grand Average  of  Hydropathicity | Relative  Hydrophobicity | Sequence  Similarity | Sequence  Similarity Index | Activity of Already Reported Peptides |
| QAEFGN | G1 | 7.86 | +1 | 665.2829 | 665.2850 | -3.16 | 4.00 | 16.67 | -1.050 | 33% | Secretolytin  QKIAEKFSGTRRG | 38.46% | Anti Gram+ |
| GVKKS | G1 | 14.01 | +1 | 518.3243 | 518.3258 | -2.89 | 10.00 | 58.00 | -0.960 | 20% | Leucrocin I  NGVQPKY | 42.86% | Anti Gram- & Gram+ |
| ENIIGG | G1 | 9.99 | +1 | 602.3111 | 602.3105 | -0.99 | 4.00 | 130.00 | 0.200 | 33% | Cathepsin G(1-5)  IIGGR | 57.14% | Anti Gram- & Gram+ |
| ASGAIK | G1 | 10.29 | +1 | 546.3196 | 546.3207 | -2.01 | 8.80 | 98.33 | 0.500 | 50% | EP5-1  ACSAG | 42.86% | Anti Gram- & Gram+ |
| PVLSFVKK | G4 | 24.05 | +1 | 917.5804 | 917.5779 | 2.72 | 10.02 | 121.25 | 0.600 | 50% | Paracentrin 1 EVASFDKSKLK | 45.45% | Anti Gram- & Gram+ |
| ATKAK | G4 | 13.67 | +1 | 518.3246 | 518.3258 | -2.32 | 10.00 | 40.00 | -0.980 | 40% | EP5-1  ACSAG | 40.00% | Anti Gram- & Gram+ |
| LLVILLV | G6 | 21.85 | +1 | 782.5709 | 782.5711 | -0.26 | 5.52 | 361.43 | 4.014 | 100% | Gageostatin A  ELLVDLL | 62.50% | Anti Gram- & Gram+ |
| KKGSG | G6 | 14.99 | +1 | 476.2779 | 476.2788 | -1.88 | 10.00 | 0.00 | -1.880 | 0% | LM12  LSKISG | 50% | Anti-Gram+ |
| AKANA | G6 | 13.59 | +1 | 474.2633 | 474.2632 | 0.21 | 8.80 | 60.00 | -0.400 | 60% | PGLa-H  KIAKVALKAL | 40% | Anti Gram- & Gram+ |
| LRLVPS | G7 | 22.40 | +1 | 685.4371 | 685.4397 | -3.79 | 9.75 | 178.33 | 0.817 | 50% | Desotamide B WLLVNG | 42.86% | Anti-Gram+ |
| SKGGK | G10 | 13.59 | +1 | 476.2781 | 476.2788 | -1.47 | 10.00 | 0.00 | -1.880 | 0% | LM12  LSKISG | 42.86% | Anti-Gram+ |

| **Supplementary Table 5 \|** *Peptides from immune-challenged desert locust nymphs* | | | | | | | | | | | | | |
| --- | --- | --- | --- | --- | --- | --- | --- | --- | --- | --- | --- | --- | --- |
| Peptide Sequence | Fraction | Retention  Time | Charge | Observed  m/z | Theoretical m/z | ΔPPM  Error | PI Value | Aliphatic  Index | Grand Average  of  Hydropathicity | Relative  Hydrophobicity | Sequence Similarity | Similarity Index | Activity of Already Reported Peptides |
| KAQAG | F8 | 13.64 | +1 | 474.2635 | 474.2632 | 0.633 | 8.75 | 40.00 | -0.840 | 40% | EP5-1  ACSAG | 50% | Anti Gram- & Gram+ |
| TYSFAVQLQTKEGY | F8 | 8.44 | +1 | 1635.7976 | 1635.8055 | -4.829 | 5.66 | 55.71 | -0.500 | 29% | Lacrain RYPAVGYT | 33.33% | Anti-Gram- |
| LKPHA | F8 | 15.86 | +1 | 566.3474 | 566.3451 | 4.06 | 8.76 | 98.00 | -0.620 | 40% | Clovibactin  FLKSNALL | 37.5% | Anti Gram- & Gram+ |
| NVKGG | F8 | 13.83 | +1 | 474.263 | 474.2632 | 0.63 | 8.75 | 58.00 | -0.800 | 20% | Brevifactin  PLGG  Plantaricin ZJ008  QWGGG | 40% | Anti-Gram+ & Gram- |
| ILLIIAF | F8 | 20.79 | +1 | 802.5365 | 802.5398 | -4.11 | 5.52 | 292.86 | 3.671 | 100% | PaDBS1R6F10  KKLRLKIAFK | 50% | Anti-Gram+ & Gram- |
| LIPLLGI | F8 | 19.09 | +1 | 738.5080 | 738.5085 | -0.677 | 5.52 | 278.57 | 2.629 | 71% | MDN-0066  LETLLSLI | 50% | Anti-Gram+ & Gram- |
| GSGST | F8 | 6.44 | +1 | 409.1718 | 409.1719 | -0.24 | 5.52 | 0.00 | -0.620 | 0% | Cyclopetide 1  GSPE | 40% | Anti-Gram+ |
| GNKGV | F8 | 13.97 | +1 | 474.2635 | 474.2632 | 0.63 | 8.75 | 58.00 | -0.800 | 20% | Plantaricin SLG1  YGNGVFSVIK | 36.36% | Anti-Gram+ & Gram- |
| IFGMSFFMNVKEKSG | F8 | 8.59 | +1 | 1721.8379 | 1721.8350 | 1.684 | 8.59 | 45.33 | 0.247 | 37.5% | IsCT2 IFGAIWNGIKSLF | 43.75% | Anti-Gram+ & Gram- |
| VKILG | F8 | 23.31 | +1 | 529.3696 | 529.3669 | 5.10 | 8.72 | 214.00 | 1.640 | 60% | Desotamide E  WLVING | 42.86% | Anti-Gram+ |
| MNELMRLAHEFL | F8 | 8.23 | +1 | 1503.7355 | 1503.7407 | -3.458 | 5.38 | 105.83 | 0.133 | 58% | HYL GIMSSLMKKLAAHIAK | 37.5% | Anti-Gram+ & Gram- |
| LPLLLVL | F8 | 20.40 | +1 | 780.5549 | 780.5554 | -0.64 | 5.52 | 320.00 | 3.086 | 86% | MDN-0066-beta  LETLLSLV | 55.56% | Anti-Gram+ & Gram- |
| TSATS | F8 | 7.12 | +1 | 467.2141 | 467.2113 | 5.99 | 5.19 | 20.00 | -0.240 | 20% | Pentapeptide TLSAC | 50% | Anti-Gram+ & Gram- |
| AKVLV | F8 | 22.98 | +1 | 529.3696 | 529.3669 | 5.10 | 8.80 | 214.00 | 2.020 | 80% | Baceridin  WAIVLL | 50% | Anti-Gram+ |
| AKVIV | F8 | 23.08 | +1 | 529.3696 | 529.3669 | 5.10 | 8.80 | 214.00 | 2.160 | 80% | EP2  AMVSS | 40% | Anti-Gram+ & Gram- |
| EYFKQ | F8 | 8.017 | +1 | 715.3419 | 715.3452 | -4.61 | 6.10 | 0.00 | -1.880 | 20% | R-Thanatin  IYNCRRRFCKQR | 33.33% | Anti-Gram+ |
| HPSFSFGDKTEAEKIS | F8 | 8.48 | +1 | 1779.8458 | 1779.8509 | -2.87 | 5.45 | 30.63 | -0.919 | 25% | Meucin-18  FFGHLFKLATKIIPSLFQ | 34.78% | Anti-Gram+ & Gram- |
| PIIIRRG | F8 | 20.87 | +1 | 824.5466 | 824.5426 | 4.85 | 12.00 | 167.14 | 0.357 | 43% | PM15  PIIYCNRRTGKCQRM | 40% | Anti-Gram+ & Gram- |
| SSASG | F8 | 6.71 | +1 | 409.1718 | 409.1695 | 5.62 | 5.24 | 20.00 | -0.200 | 20% | EP5-1  ACSAG | 50% | Anti-Gram+ & Gram- |
| LIVKG | F8 | 23.03 | +1 | 529.3696 | 529.3669 | 5.10 | 8.72 | 214.00 | 1.640 | 60% | Desotamide B  WLLVNG | 50% | Anti-Gram+ |
| APPSLN | F8 | 16.09 | +1 | 599.3216 | 599.3189 | 4.51 | 5.57 | 81.67 | -0.317 | 33% | RLID 12.1  TPPQS | 42.86% | Anti-Gram+ & Gram- |
| QHETSL | F8 | 8.10 | +1 | 715.3419 | 715.3411 | 1.12 | 5.24 | 65.00 | -1.317 | 17% | MDN-0066-beta  LETLLSLV | 44.44% | Anti-Gram+ & Gram- |
| LPLLLVL | F5 | 20.57 | +1 | 780.5551 | 780.5554 | -0.38 | 5.52 | 320.00 | 3.086 | 86% | MDN-0066-beta  LETLLSLV | 55.56% | Anti-Gram+ & Gram- |
| AAGQK | F5 | 13.96 | +1 | 474.2633 | 474.2632 | 0.21 | 8.80 | 40.00 | -0.840 | 40% | EP5-1  ACSAG | 42.86% | Anti-Gram+ & Gram- |
| EPKTMFWRDHSGR | F5 | 8.22 | +1 | 1647.7791 | 1647.7851 | -3.64 | 8.85 | 0.00 | -1.754 | 23% | msHemerycin  SVEIPKPFKWNDSF | 41.18% | Anti-Gram+ & Gram- |
| LIPLLGI | F5 | 19.45 | +1 | 738.5083 | 738.5085 | -0.27 | 5.52 | 278.57 | 2.629 | 71% | MDN-0066  LETLLSLI | 50% | Anti-Gram+ & Gram- |
| KATKA | F5 | 14.07 | +1 | 518.3242 | 518.3258 | -3.09 | 10.00 | 40.00 | -0.980 | 40% | PGLa-H  KIAKVALKAL | 40% | Anti-Gram+ & Gram- |
| SGVKK | F5 | 14.09 | +1 | 518.3248 | 518.3258 | -1.93 | 10.00 | 58.00 | -0.960 | 20% | Delftibactin-A  SDTGTKSRK | 44.44% | Anti-Gram+ & Gram- |
| VHDLQGWLSDKM | F5 | 8.23 | +1 | 1429.6975 | 1429.6934 | 2.87 | 5.21 | 89.17 | -0.500 | 42% | JCpep7  KVFLGLK | 38.46% | Anti-Gram+ & Gram- |
| SNLGQ | F5 | 9.02 | +1 | 518.2530 | 518.2530 | -0.19 | 5.24 | 78.00 | -0.880 | 20% | Brevifactin  PLGG | 40% | Anti-Gram+ & Gram- |
| SSASG | F5 | 6.43 | +1 | 409.1695 | 409.1695 | 5.38 | 5.24 | 20.00 | -0.200 | 20% | EP5-1  ACSAG | 50% | Anti-Gram+ & Gram- |
| SKPVGG | F5 | 13.15 | +1 | 544.3050 | 544.3050 | -1.29 | 8.47 | 48.33 | -0.483 | 17% | Brevifactin  PLGG | 50% | Anti-Gram+ & Gram- |
| ATKAK | F5 | 14.08 | +1 | 518.3258 | 518.3258 | -3.09 | 10.00 | 40.00 | -0.980 | 40% | EP5-1  ACSAG | 40% | Anti-Gram+ & Gram- |
| YSTYIVG | F3 | 7.69 | +1 | 803.3945 | 803.3976 | -3.86 | 5.52 | 97.14 | 0.600 | 29% | Humimycin B  YSYYTIV | 62.5% | Anti-Gram+ |
| FFTYSLQFYVPF | F3 | 8.45 | +1 | 1559.7633 | 1559.7611 | 1.41 | 5.52 | 56.67 | 0.833 | 50% | Megin 1  FLKGCWTKWYSLKPKCPF | 38.89% | Anti-Gram+ & Gram- |
| ATQVK | F3 | 12.49 | +1 | 546.319 | 546.3207 | -3.11 | 8.80 | 78.00 | -0.420 | 40% | S. amritsarensis lipopeptide  ATQSHQ | 50% | Anti-Gram+ |
| IRDQQN | F3 | 8.23 | +1 | 773.3837 | 773.3861 | -3.10 | 5.84 | 65.00 | -2.333 | 17% | S. marcescens bacteriocin  MRTGN | 33.33% | Anti-Gram- |
| MNELMRLAHEFL | F3 | 8.46 | +1 | 1503.7477 | 1503.7407 | 4.66 | 5.38 | 105.83 | 0.133 | 58% | (I5,R8) Mastoparan, Leu-Rich  INLKILARLAKKIL | 42.86% | Anti-Gram+ & Gram- |
| EFHELA | F3 | 7.43 | +1 | 745.3516 | 745.3476 | 5.37 | 4.51 | 81.67 | -0.300 | 50% | Plicatamide  FFHLHFH | 37.5% | Anti-Gram+ & Gram- |
| AMPPAL | F3 | 16.57 | +1 | 599.3212 | 599.3182 | 5.00 | 5.57 | 98.33 | 1.017 | 67% | Peptide 7  APPPGLSAGV | 40% | Anti-Gram+ & Gram- |
| RMMKTH | F3 | 7.60 | +1 | 803.3945 | 803.3976 | -3.85 | 11.00 | 0.00 | -1.417 | 33% | RP9  RGSALTHLP | 33.33% | Anti-Gram+ & Gram- |
| YFWLS | F3 | 7.93 | +1 | 715.3412 | 715.3411 | 0.14 | 5.52 | 78.00 | 0.720 | 60% | Temporin-SHf  FFFLSRIF | 37.5% | Anti-Gram+ & Gram- |
| ASGAIK | F3 | 12.51 | +1 | 546.3195 | 546.3207 | -2.19 | 8.80 | 98.33 | 0.500 | 50% | EP5-1  ACSAG | 42.86% | Anti-Gram+ & Gram- |
| LRFYP | F3 | 8.97 | +1 | 696.3956 | 696.3956 | 0.00 | 8.75 | 78.00 | -0.160 | 40% | PAF44  LRKKWFW | 37.5% | Anti-Gram+ & Gram- |
| TPLDKARERSDEG | F3 | 8.59 | +1 | 1473.7227 | 1473.7253 | -1.76 | 4.78 | 37.69 | -1.908 | 15% | PvAMP164  RSVLKAHCRICRRRG | 35.29% | Anti-Gram- |
| KPVSRV | F1 | 22.58 | +1 | 685.4357 | 685.4316 | 5.98 | 11.00 | 96.67 | -0.400 | 33% | SyCPA 4  AKSKRKGV | 40% | Anti-Gram+ |
| VILRQG | F1 | 24.26 | +1 | 685.4357 | 685.4316 | 5.98 | 9.72 | 178.33 | 0.683 | 50% | Cn-AMP1  SVAGRAQGM | 44.44% | Anti-Gram+ & Gram- |
| RVILQG | F1 | 24.23 | +1 | 685.4357 | 685.4316 | 5.98 | 9.72 | 178.33 | 0.683 | 50% | F1  LRVRRTLQ | 44.44% | Anti-Gram- |
| YFWLS | F1 | 7.89 | +1 | 715.3411 | 715.3411 | 0.00 | 5.52 | 78.00 | 0.720 | 60% | Temporin-SHf  FFFLSRIF | 37.5% | Anti-Gram+ & Gram- |
| ILVHPLFS | F1 | 16.67 | +2 | 463.2769 (926.5538) | 926.5500 | 4.10 | 6.74 | 182.50 | 1.688 | 63% | Temporin-1KM  FIPLVSGLFSRLL | 46.15% | Anti-Gram+ & Gram- |
| LGRNLI | F1 | 23.69 | +1 | 685.4357 | 685.4316 | 5.98 | 9.75 | 195.00 | 0.617 | 50% | Ctry2459  FLGFLKNLF | 44.44% | Anti-Gram+ & Gram- |
| RVNLLA | F1 | 22.84 | +1 | 685.4357 | 685.4316 | 5.98 | 9.75 | 195.00 | 0.933 | 67% | M5  KIRVRLSA | 50% | Anti-Gram- |
| EFTFFL | F1 | 7.74 | +1 | 803.3941 | 803.3935 | 0.75 | 4.00 | 65.00 | 1.333 | 67% | SyCPA 123  FSTRF | 42.86% | Anti-Gram+ & Gram- |
| EENRKQ | F1 | 7.62 | +1 | 803.3941 | 803.3967 | -3.24 | 6.24 | 0.00 | -3.733 | 0% | R-Thanatin  IYNCRRRFCKQR | 33.33% | Anti-Gram+ |
| ALAVVRG | F1 | 22.49 | +1 | 685.4357 | 685.4316 | 5.98 | 9.79 | 167.14 | 1.557 | 71% | Bac2A G12  RLARIVVIRVAG | 50% | Anti-Gram+ & Gram- |
| RKLTAP | F1 | 23.11 | +1 | 685.4357 | 685.4316 | 5.98 | 11.00 | 81.67 | -0.850 | 33% | PAF26.f5a  RKKWAW | 50% | Anti-fungal |
| QFFIQLRREPSEDYD  TLLGFNMTTYRSS | F1 | 8.48 | +2 | 1707.8307 (3415.6614) | 3415.6417 | 5.76 | 4.78 | 55.71 | -0.746 | 29% | Nisin Z  ITSISLCTPGCKTGALMGCNMKTATCNCSIHVSK | 31.43% | Anti-Gram+ |
| VRLAGGL | F1 | 23.05 | +1 | 685.4357 | 685.4316 | 5.98 | 9.72 | 167.14 | 1.186 | 57% | JCpep7  KVFLGLK | 44.44% | Anti-Gram+ & Gram- |
| ILLRNG | F1 | 24.44 | +1 | 685.4357 | 685.4316 | 5.98 | 9.75 | 195.00 | 0.617 | 50% | Dosotamide A  WLLING | 66.67% | Anti-Gram+ |
| LGRNLI | F1 | 24.43 | +1 | 685.4357 | 685.4316 | 5.98 | 9.75 | 195.00 | 0.617 | 50% | Ctry2459  FLGFLKNLF | 44.44% | Anti-Gram+ & Gram- |
| EFHELA | F1 | 7.31 | +1 | 745.3505 | 745.3476 | 3.89 | 4.51 | 81.67 | -0.300 | 50% | Plicatamide  FFHLHFH | 37.5% | Anti-Gram+ & Gram- |
| LFENPPS | F1 | 7.63 | +1 | 803.3941 | 803.3895 | 5.72 | 4.00 | 55.71 | -0.629 | 29% | Neurotensin  QLYENKPRRPYIL | 38.46% | Anti-Gram+ & Gram- |
| GLLLNR | F1 | 22.75 | +1 | 685.4357 | 685.4316 | 5.98 | 9.75 | 195.00 | 0.617 | 50% | Dosotamide A  WLLING | 50% | Anti-Gram+ |
| IANVLR | F1 | 22.47 | +1 | 685.4357 | 685.4316 | 5.98 | 9.75 | 195.00 | 1.050 | 67% | Desotamide B  WGNVLL | 50% | Anti-Gram+ & Gram- |
| TALRKP | F1 | 23.71 | +1 | 685.4357 | 685.4316 | 5.98 | 11.00 | 81.67 | -0.850 | 33% | CopN5(22–30)  LHCIALRKK | 44.44% | Anti-Gram- |
| NPQSRN | F1 | 8.01 | +1 | 715.3411 | 715.3443 | -4.47 | 9.75 | 0.00 | -2.900 | 0% | RLID 12.1  TPPQS | 42.86% | Anti-Gram+ & Gram- |
| QVLKDYYFFNMGDFG  GVMVAVKYYKAQG | F13 | 8.41 | +2 | 1619.7887  (3239.5774) | 3239.5735 | 1.20 | 8.30 | 62.50 | 0.014 | 43% | Ranatuerin-1C  SMLSVLKNLGKVGLGLVACKINKQC | 37.5% | Anti-Gram+ & Gram-, Anti-fungal |
| WQYGRWVDVVIDDR | F13 | 9.12 | +1 | 1807.8816 | 1807.8916 | -5.53 | 4.43 | 90.00 | -0.671 | 43% | Bac2A sub5  RRWKIVVIRWRR | 40% | Anti-Gram+ & Gram-, Anti-fungal |
| WDRFQSRAYSL | F13 | 8.49 | +1 | 1429.6952 | 1429.7013 | -4.26 | 8.75 | 44.55 | -1.036 | 36% | RetroLL-10+  KWKRFFRGWL | 41.67% | Anti-Gram+ & Gram- |
| WFGDWSDGALFQVG  REFTNRVDLERPSWK | F13 | 8.86 | +2 | 1749.8574 (3499.7148 | 3499.6972 | 5.02 | 4.86 | 50.34 | -0.810 | 38% | Buforin II  TRSSRAGLQFPVGRVHRLLRK | 35.48% | Anti-Gram+ & Gram- |
| EENRKQ | F13 | 7.60 | +1 | 803.3950 | 803.3967 | -2.12 | 6.24 | 0.00 | -3.733 | 0% | Y12M  YCNRRTGKCQRM | 33.33% | Anti-Gram+ & Gram-, Anti-fungal |
| AGRIF | F2 | 14.90 | +1 | 564.3309 | 564.3294 | 2.66 | 9.79 | 98.00 | 0.840 | 60% | PvAMP82  GRIFRLLRK | 40% | Anti-Gram- |
| ASGAIK | F2 | 12.78 | +1 | 546.3195 | 546.3207 | -2.19 | 8.80 | 98.33 | 0.500 | 50% | EP5-1  ACSAG | 42.86% | Anti-Gram+ & Gram- |
| MAAHEFDARRRMIS | F2 | 8.42 | +1 | 1691.8246 | 1691.8259 | -0.76 | 9.28 | 49.29 | -0.571 | 50% | Pinipesin  VAEARQGSFSY | 33.33% | Anti-Gram+ & Gram-, Anti-fungal |
| LYDDFMSRAKTR | F2 | 8.26 | +1 | 1503.7362 | 1503.7415 | -3.52 | 8.59 | 40.83 | -1.033 | 33% | Temporin-WY2  FLPFLASLALKIL | 33.33% | Anti-Gram+ & Gram- |
| RSVLREWYTHNPY | F2 | 8.74 | +1 | 1721.8557 | 1721.8548 | 0.52 | 8.60 | 52.31 | -1.369 | 23% | Lysocin E  TRSGFLREQWIT | 43.75% | Anti-Gram+ |
| KYYTGN | F2 | 7.30 | +1 | 745.3516 | 745.3476 | 5.36 | 8.50 | 0.00 | -1.850 | 0% | S. marcescens bacteriocin  MRTGN | 50% | Anti-Gram- |
| AEHQPPAQDKRDVSQG | F2 | 8.80 | +1 | 1763.8491 | 1763.8461 | 1.70 | 5.38 | 30.63 | -1.825 | 19% | Peptide 8  APPPGYAMESDSFS | 33.33% | Anti-Gram+ & Gram- |
| ASGAIK | F4 | 12.48 | +1 | 546.3199 | 546.3207 | -1.46 | 8.80 | 98.33 | 0.500 | 50% | EP5-1  ACSAG | 42.86% | Anti-Gram+ & Gram- |
| KQAAG | F4 | 13.75 | +1 | 474.2632 | 474.2632 | 0.00 | 8.75 | 40.00 | -0.840 | 40% | Emericellipsin A  PQAAIVASG | 44.44% | Anti-Gram+ & Gram-, Anti-fungal |
| NVKGG | F4 | 13.99 | +1 | 474.2632 | 474.2632 | 0.00 | 8.75 | 58.00 | -0.800 | 20% | Brevifactin  PLGG | 40% | Anti-Gram+ & Gram-, Anti-fungal |
| YGGYLNYTVRYVPA | F4 | 8.25 | +1 | 1635.8035 | 1635.8126 | -5.56 | 8.43 | 76.43 | -0.164 | 29% | PG-1 analog 2  RGGRLVYTRPRFTVIVGR | 38.89% | Anti-Gram+ & Gram- |
| VKGGN | F4 | 13.71 | +1 | 474.2632 | 474.2632 | 0.00 | 8.75 | 58.00 | -0.800 | 20% | Plantaricin ZJ008  QWGGG | 40% | Anti-Gram+ & Gram- |
| AKANA | F4 | 13.68 | +1 | 474.2632 | 474.2632 | 0.00 | 8.80 | 60.00 | -0.400 | 60% | PGLa-H  KIAKVALKAL | 40% | Anti-Gram+ & Gram- |
| QNRQID | F4 | 8.26 | +1 | 773.3838 | 773.3861 | -2.97 | 5.84 | 65.00 | -2.333 | 17% | GP-2B  CND | 33.33% | Anti-Gram+, |
| GNYENINWEYYGA  YFRNLISLFGHIVD | F4 | 8.45 | +2 | 1634.7828 (3269.5656) | 3269.5562 | 2.88 | 4.65 | 86.67 | -0.281 | 37% | Caerin 3.3  GLWEKIKEKANELVSGIVEGVK | 35.85% | Anti-Gram+ & Gram- |
| HVQRSDMNKLIM  NYLVTEGFKEATEKFQQE | F11 | 8.92 | +2 | 1807.8868 (3615.7736) | 3615.7806 | -1.94 | 5.59 | 61.67 | -0.887 | 33% | TeRu3  AVLSFVHKLFLNFLHVDTSKGKCRATLQ | 38.24% | Anti-Gram- |
| LIVKG | F11 | 23.20 | +1 | 529.3698 | 529.3669 | 5.48 | 8.75 | 214.00 | 1.640 | 60% | Dosotamide B  WLLVNG | 50% | Anti-Gram+ |
| VKILG | F11 | 23.15 | +1 | 529.3698 | 529.3669 | 5.48 | 8.75 | 214.00 | 1.640 | 60% | Dosotamide E  WLVING | 42.86% | Anti-Gram+ |
| QRDSLGN | F11 | 7.75 | +1 | 789.3788 | 789.3810 | -2.79 | 5.84 | 55.71 | -1.771 | 14% | S. marcescens bacteriocin  MRTGN | 42.86% | Anti-Gram- |
| AKVLV | F11 | 22.99 | +1 | 529.3698 | 529.3669 | 5.48 | 8.80 | 214.00 | 2.020 | 80% | Baceridin  WAIVLL | 50% | Anti-Gram+ |
| KLVLG | F11 | 23.07 | +1 | 529.3698 | 529.3669 | 5.48 | 8.75 | 214.00 | 1.500 | 60% | Dosotamide E  WLVING | 50% | Anti-Gram+ |
| EAFYLF | F11 | 7.81 | +1 | 789.3788 | 789.3778 | 1.26 | 4.00 | 81.67 | 1.067 | 67% | PL-135  FHFHLHF | 42.86 | Anti-Gram+ & Gram- |
| ASGELRKHSTSPFPY | F11 | 8.84 | +1 | 1677.8301 | 1677.8385 | -5.01 | 8.64 | 32.67 | -0.980 | 20% | Pinipesin  VAEARQGSFSY | 37.5% | Anti-Gram+ & Gram-, Anti-fungal |
| ALPQQQQQQQQQQQQ | F11 | 8.71 | +1 | 1837.8955 | 1837.8941 | 0.76 | 5.57 | 32.67 | -2.533 | 13% | Alpha-basrubrin  GADFQECMKEHSQKQHQHQG | 30% | Anti-viral, Anti-fungal |
| LVYWQEPQLPNEM | F11 | 8.45 | +1 | 1647.7948 | 1647.7877 | 4.31 | 3.79 | 82.31 | -0.708 | 38% | Tigerinin 3  RVCYAIPLPICY | 35.71% | Anti-Gram+ & Gram-, Anti-fungal |
